# Supplementary material for: Path2Omics: Enhanced transcriptomic and methylation prediction accuracy from tumor histopathology
Source: bioRxiv. 2025 Mar 16:2025.02.26.640189. Preprint. [Version 2] doi: 10.1101/2025.02.26.640189 (PMC12190742; doi:10.1101/2025.02.26.640189)
Supplement: Supplement 1 [file NIHPP2025.02.26.640189v2-supplement-1.pdf]

bioRxiv preprint doi: https://doi.org/10.1101/2025.02.26.640189; this version posted March 16, 2025. The copyright holder for this preprint (which was not certified by peer review) is the author/funder. This article is a US Government work. It is not subject to copyright under 17 USC 105 and is also made available for use under a CC0 license.

Additional information

Supplementary Information

Supplementary Table 1. Number of slides and patients used in each TCGA cohort for Path2Omics model development for predicting gene expression

No.	Cohort	# FFPE slides	# FF slides	# Patients
1	BRCA	1,096	1,487	1,034
2	UCEC	558	696	498
3	THCA	510	524	497
4	KIRC	496	991	494
5	LGG	781	627	465
6	LUSC	490	695	456
7	LUAD	504	706	442
8	HNSC	437	611	416
9	PRAD	429	487	383
10	BLCA	443	399	380
11	COAD	371	694	367
12	LIHC	371	379	357
13	STAD	294	393	273
14	KIRP	281	341	261
15	CESC	270	268	260
16	SARC	593	252	252
17	PCPG	194	176	176
18	PAAD	195	202	175
19	TGCT	253	155	153

bioRxiv preprint doi: https://doi.org/10.1101/2025.02.26.640189; this version posted March 16, 2025. The copyright holder for this preprint (which was not certified by peer review) is the author/funder. This article is a US Government work. It is not subject to copyright under 17 USC 105 and is also made available for use under a CC0 license.

20	READ	138	278	138
21	ESCA	120	118	118
22	THYM	170	116	116
23	GBM	201	173	86
	Total	9,195	10,768	7,797

Supplementary Table 2. Number of slides and patients used in each TCGA cohort for Path2Omics model development for predicting DNA methylation

No.	Cohort	# FFPE slides	# FF slides	# Patients
1	BRCA	800	951	746
2	UCEC	431	471	387
3	THCA	515	529	502
4	KIRC	287	541	287
5	LGG	807	644	476
6	LUSC	342	440	329
7	LUAD	436	597	390
8	HNSC	465	645	443
9	PRAD	436	494	390
10	BLCA	447	403	384
11	COAD	225	380	221
12	LIHC	377	385	363
13	STAD	333	428	308
14	KIRP	263	307	248
15	CESC	272	270	262
16	SARC	596	254	254

bioRxiv preprint doi: https://doi.org/10.1101/2025.02.26.640189; this version posted March 16, 2025. The copyright holder for this preprint (which was not certified by peer review) is the author/funder. This article is a US Government work. It is not subject to copyright under 17 USC 105 and is also made available for use under a CC0 license.

17	PCPG	195	177	177
18	PAAD	202	215	182
19	TGCT	253	155	153
20	READ	74	138	74
21	ESCA	141	139	139
22	THYM	175	121	121
23	GBM	87	91	52
	Total	8,159	8,775	6,888

Supplementary Table 3. Number of slides and patients in each external cohort for Path2Omics model evaluation for predicting gene expression

No.	Cohort	# Slides	# Patients	Slide type
1	NCI-LGG	309	307	FFPE
2	CPTAC-BRCA	106	106	FFPE
3	CPTAC-KIRC	312	221	FFPE
4	CPTAC-LUSC	109	108	FFPE
5	CPTAC-LUAD	224	224	FFPE
6	CPTAC-COAD	103	103	FFPE
7	TransNeo-BRCA	160	160	FF
	Total	1,323	1,229	

bioRxiv preprint doi: https://doi.org/10.1101/2025.02.26.640189; this version posted March 16, 2025. The copyright holder for this preprint (which was not certified by peer review) is the author/funder. This article is a US Government work. It is not subject to copyright under 17 USC 105 and is also made available for use under a CC0 license.

Supplementary Table 4. Number of patients in each TCGA cohort for survival analysis based on the predicted gene expression and DNA methylation

No.	Cohort	Gene expression	DNA methylation
1	BRCA	1,022	739
2	THCA	495	500
3	KIRC	489	284
4	LUSC	446	320
5	LUAD	426	377
6	BLCA	377	381
7	HNSC	373	393
8	COAD	355	211
9	LIHC	333	339
10	STAD	263	298
11	KIRP	236	228
12	ESCA	111	132
	Total	4,928	4,202
